# Is It Necessary to Terminate Pregnancy before Hydatid Cyst Surgery?

**Published:** 2020

**Authors:** Azar Danesh SHAHRAKI, Azam ZAFARBAKHSH, Amirreza FARHADIAN DEHKORDI

**Affiliations:** 1. Department of Obstetrics and Gynecology, School of Medicine, Isfahan University of Medical Sciences, Isfahan, Iran; 2. Department of General Medicine, School of Medicine, Isfahan University of Medical Sciences, Isfahan, Iran

## Dear Editor-in-Chief

Hydatid cyst is a rare phenomenon that occurs in about 1/20000–1/30000 of pregnancies (1). It appears at the larval stage of *Echinococcus multilocularis* or *E. granulosus*. There is, however, no standard treatment during pregnancy. Hydatid cyst disease is life-threatening for the mother/fetus (1). It is diagnosed during pregnancy by serological testing and ultrasonography. Clinical manifestations of the disease depend on the size and location of cysts. Treatment modalities are surgery and drug therapy.

Surgery is the choice treatment for complicated hydatid cyst but replacement rate may be widely variable. Medicinal treatment with albendazole is prescribed for non-pregnant and asymptomatic patients, which may take several years to yield results. However, albendazole, classified at C Group of the Food and Drug Administration (FDA), has a teratogenic potential for the fetus(2).

The main goal of this article is to answer the following question: Is pregnancy termination a perquisite of surgery? What is the best way of managing pregnant patient with hydatid cyst?

We did an extensive review of relevant literature but could not find any article about termination of pregnancy before surgery or medical treatment of hydatid cyst disease.

In a multi-center retrospective study, 24 pregnant women with complicated hepatic hydatid cyst and gestational age of 5–29 weeks were treated surgically(1). They reported 21 full-term live births, one spontaneous abortion, and two neonatal deaths without any maternal deaths. Another study reported surgical treatment of 2 pregnant women with a gestational age of 13 and 23 weeks for uncomplicated hepatic hydatid disease (1, 3). Surgical drainage was performed for 7 pregnant patient with a gestational age of 13–24 weeks (4).

Percutaneous treatment with oral albendazol before and after procedure was investigated in 13-week pregnant woman for multiple hepatic and splenic hydatid cysts (5).

Sobhani et al. also reported symptoms of anaphylactic shock caused by intraperitoneal rupture of hydatid cyst in 11-week pregnant woman, who had to undergo surgery and then medically treated with albendazole (6). A case of conservative management in pregnancy was reported in a 25-week pregnant woman with the rupture of lung the pulmonary hydatid cysts without any medical management, who had cesarean-section at week 34 and put on oral albendazol 6 weeks after delivery (7).

**Fig. 1:**
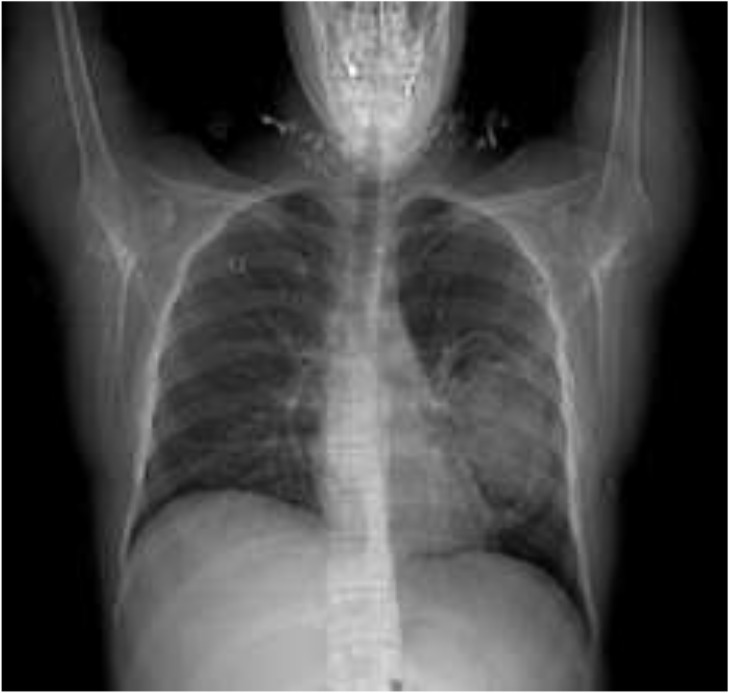
CXR; the lung large hydatid cyst is in left side

**Fig. 2:**
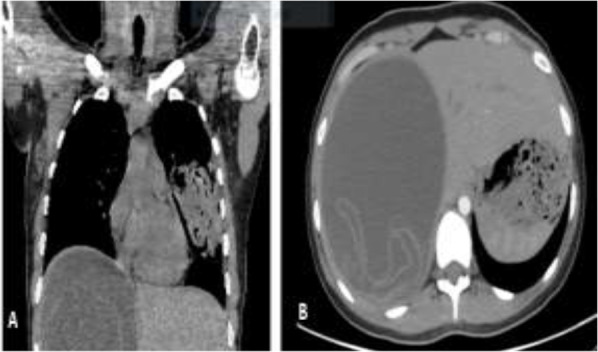
A, Coronal CT scan; Hydatid cysts in liver and the left lung. B, Axial CT scan; large hydatid cyst in the liver

We examined a 26-year-old nulligravida woman with a known case of liver and lung hydatid cysts who had an unplanned pregnancy referred to the hospital. During the pregnancy, the patient was monitored for symptoms of the illness. She had severe and frequent coughing. During the first two months of pregnancy, the lung large cyst ruptured, causing the enlargement of cysts size (Fig. 1, 2). She was recommended to have an abortion before surgery.

After abortion, she was treated with thoracotomy and laparotomy simultaneously. The cysts were evacuated and treatment with albendazole was initiated. The results of 2-year follow-up suggest that she has no signs or symptoms.

Finally, we emphasize that in previous studies and WHO Staging, we did not find any recommendation to terminate a pregnancy before treating a hydatid cyst.

However, the question is “what signs/symptoms or the disease stages do call for early termination of pregnancy?” Should termination really occur in a pregnant woman with hydatid cyst? Surgery may cure the patient, but broad replacement rate is a problem at the reproductive age. Given the above, there is still debate about proper procedure for the treatment and management of a pregnant patient with hydatid cysts.

## References

[1] Baraket O Triki W Rebii S Management of Complicated Hydatid Cyst in Pregnancy. A Multicenter Study. Hell J Surg. 2018;90(4):172–6.

[2] US Food and Drug Administration Prescribing Information Albenza. 2009;4–11. https://www.accessdata.fda.gov/drugsatfda_docs/label/2009/020666s005s006lbl.pdf

[3] Ercetin C Ozden I Iyibozkurt C Hepatic hydatid disease requiring urgent treatment during pregnancy. Ulus Travma Acil Cerrahi Derg. 2013;19(2):119–22. 23599194 10.5505/tjtes.2013.21548

[4] Bakdik S Arslan S Oncu F. Long-term results of percutaneously treated multiple hepatic and splenic hydatid cysts in a pregnant woman. J Infect Dev Ctries. 2018;12(8):680–682. 31958333 10.3855/jidc.10104

[5] Sobhani R Alesaeidi S Mahmoudabadi A. Intraabdominal Ruptured Hydatid Cyst in a Pregnant Woman during First Trimester. Medical Journal of Mashhad University of Medical Sciences. 2013;56(2):123–6.

[6] Al-Ani A Elzouki A Mazhar R. An Imported Case of Echinococcosis in a Pregnant Lady with Unusual Presentation. Case Rep Infect Dis. 2013;2013:753848. 23401812 10.1155/2013/753848 PMC3562643

